# Moments of Care: Perceptions of Young Carers and Day-to-Day Well-Being

**DOI:** 10.3390/healthcare13030292

**Published:** 2025-01-31

**Authors:** Melinda S. Kavanaugh, Matthew J. Zawadzki, Kayla T. Johnson, Miranda R. Boville

**Affiliations:** 1 Helen Bader School of Social Welfare, University of Wisconsin-Milwaukee, Milwaukee, WI 53211, USA; 2 Department of Psychological Sciences, University of California Merced, Merced, CA 95343, USA; 3 Division of Epidemiology and Community Health, University of Minnesota-Twin Cities, Minneapolis, MN 55454, USA; 4 Medical College of Wisconsin, Milwaukee, WI 53226, USA

**Keywords:** young carers, caregiving youth, EMA, family caregiving, ALS

## Abstract

**Background/Objectives:** Over 5 million youth under the age of 19 provide daily, hands-on care to an ill or injured family member across the United States. Yet how these young carers perceive the care they deliver in the moment, and how these perceptions relate to well-being, is unexplored, particularly in complex neurological conditions. This paper presents initial data on young carers for a family member with amyotrophic lateral sclerosis (ALS). **Methods:** Ecological momentary assessment (EMA) was used to measure perceptions of care in the moments of care and the cognitive and emotional states of the young carers during those moments. Young carers (*n* = 15) aged 10–19 were followed for seven days, completing assessments three times per day, which provided 260 total measurements. Young carers reported frequently engaging in caregiving (~39% of assessments). **Results:** The results indicated that it was not simply performing a caregiving task that related to outcomes, but rather how caregiving moments were perceived that mattered. Caregiving moments perceived as more fulfilling resulted in young carers feeling less discontent and more focused, whereas caregiving moments perceived as lacking resources predicted more discontent and distress. Exploratory analyses highlighted the potential for burden for young carers. They reported high levels of worry when they were not around the care recipient, with this worry predicting feeling more discontent and distressed. **Conclusions:** Young carers are deeply involved in care and perceive care differently across moments, both positive and negative. These initial data can be used to develop targeting support programs in the moment of care, potentially lessening the negative impacts of care.

## 1. Introduction

Known as “young carers”, over 5 million children and youth under the age of 19 provide care to an ill or injured family member across the United States [1], including amyotrophic lateral sclerosis (ALS) [2]. These young carers engage in a range of care tasks, including bathing, feeding, toileting, and transferring. Few, if any, care tasks undertaken by adults are not engaged in by youth, underscoring the intense role young carers play as caregivers throughout the day, both before and after school, and into the evening [2,3,4]. Yet how care in the moment is perceived and whether care impacts momentary well-being is unclear.

Young carers are the most underacknowledged and unaddressed caregiving population in the United States [3,5], with the overwhelming attention in both programming and research paid to adults as caregivers. Described as “young carers” in the European context [6], and “caregiving youth” [4] and “young caregivers” [1] in the United States, the lack of a consistent nomenclature may add to the lack of awareness and programming [7]. The lack of consistent description, and omittance in national care programs, has created gaps in knowledge of the overall experience of being a young carer, including what happens in the moment of care, both positive and negative. Using ecological momentary assessment (EMA) data from a sample of young carers providing care for a family member with ALS, this paper details cognitive and emotional responses young carers have while delivering recent care experiences. Beyond just whether caregiving was performed, this paper considers how caregiving moments were perceived by young carers—both in terms of whether resources are available for the care task and the derived fulfillment from it—and how these perceptions relate to cognitive and emotional well-being.

### 1.1. The Consequences of Caregiving

Research on young carers has identified the potential for a negative overall impact of care on young carers, specifically mental health impacts [8,9], underscoring the need for not only more understanding of mental health consequences but the need for targeted interventions by professionals [8,10] and wraparound family support. Moreover, due to the timing and (often) intensity of caregiving tasks, caregiving impacts school performance and attendance, with many young carers missing school activities, falling asleep in class, and getting to school late [3,4,11]. Finally, it has been found that both the care recipient’s well-being and caregiving tasks, especially at night, are significant sources of worry for young carers [12].

This paper considers the importance of both negative as well as positive effects of caregiving. Research has shown that when looking at outcomes including self-compassion and subjective well-being, young carers were no different on these outcomes than non-caregiving youth [13]. Other work suggests that some young carers find benefits in caring for an ill parent and that those who find more benefits report better adjustment [14]. Recent qualitative research of young carers suggested a mixed picture with benefits in the form of early maturity and a bond with a care recipient along with negative impacts on work, focus, and socialization with peers [15]. Thus, it is important to consider the overall pattern of caregiving for youth, including how caregiving is perceived in the moments when care is being performed. Recognizing the perception of care at the moment may allow for enhanced benefits and reduction in negatives in the moments of care, reducing overall load over time.

### 1.2. Perceptions of Moments of Care

Not all caregiving moments are perceived the same, including whether one feels they have the resources needed to address the caregiving task. Young carers frequently feel positive about care yet need support and guidance to feel confident in the care task at hand [16] and reduce the potential for stress. However, young carers are rarely provided with preparation or training, instead “winging it” and guessing how best to provide care [17]. When provided the opportunity to learn from professionals, alongside other peers, young carers had a significant increase in confidence and skill, while developing “like” peer networks and reducing social isolation [18]. These data highlight that young carers have the ability and willingness to engage in care but need to have support for preparation for the task at hand. When support and guidance are not provided, young carers may feel a lack of resources within themselves to provide care, potentially leading to increased stress and anxiety. Moreover, the role of control in the caregiving experience may play a part in the overall well-being of young carers. Recent exploratory data identified the importance of perceived control in care tasks, highlighting that more care tasks and the progression of illness negatively impacted young carers' perception of control in care situations [19], underscoring the need to assess and identify the role of control and its relationship to overall well-being

Another perception that is likely to matter is the perceived benefits one receives from engaging in care. Young carers find benefits in caregiving particularly when the burden is lower, they have the resources and outside support [20], and they find satisfaction in their social support [14]. Additionally, benefits varied by task category, with higher levels of benefits found in emotional/social care, and less so in instrumental care [21]. This research points to the need to understand care-specific experiences, particularly at the point of care, and to examine how young carers find benefits within and across tasks. This is especially critical in the US context where young carers under the age of 18 are not included in any state or national care programs, thus awareness and support are limited, leaving fewer outlets for support [5,22].

### 1.3. Objectives

This paper presents results from an initial project on young carers assisting in the care of a family member living with ALS. EMA was used to measure perceptions of care, in the moments of care, and the cognitive and emotional states of the young carers close in time to when that care was engaged.

Research questions. Research questions focused on the relationship between caregiving activities and cognitive and affective well-being. Research Question 1: How often are young carers engaging in care tasks, and what is the type of care they report doing? Research Question 2: Do young carers report differences in cognitive and emotional well-being during caregiving moments versus non-caregiving moments? Finally, Research Question 3: During caregiving moments, do momentary perceptions of feeling fulfillment and/or lack of resources when caregiving predict cognitive and emotional well-being?

We also explored whether the impact of caregiving extends to non-caregiving moments. In particular, we examined the extent to which young carers worried about not being available to deliver care in moments when they were not with their typical care recipient. Findings have clear opportunity for interventions based on care type and timing.

## 2. Materials and Methods

All study procedures were approved by the principal investigator’s (MSK) university institutional review board. Young carers were recruited via flyers posted in a neurology clinic at a large academic medical center, and through postings on the local ALS Association website and newsletters. Interested patients who had a child or youth in the family under the age of 18 were instructed to contact this study’s principal investigator’s team and complete a brief screening questionnaire via phone, video conferencing, or in-person with study personnel. Participants aged 18 could contact the principal investigator directly. Families who met eligibility criteria were invited to have their child/youth participate. Participants met with study personnel prior to the start of this study (all chose online options: phone/video conferencing) during their first visit for survey completion and instructions. Verbal consent from a parent was obtained, as well as verbal assent from the young carer. Consent forms were mailed to provide copies to participants.

### 2.1. Procedures

After consent and assent were obtained, all youth participants were given instructions on how to download the RealLife Exp (LifeData, LLC, Marion, IN, USA) EMA app on their smartphones. Participants were instructed by study staff to complete a baseline well-being survey via the installed EMA software V2.8 that took approximately 8 min. Participants were then shown the questions they would be asked in the EMA and practiced going through each one. Finally, the EMA procedure was explained to them. Study staff were available (via phone and videoconferencing) to help answer any questions.

For the following seven consecutive days, the young carers were prompted three times throughout the day to complete an assessment. We chose three time points to minimize the overall burden but to capture meaningfully different time periods (e.g., morning versus evening) in which different caregiving activities might be needed and/or different groupings of other individuals might also be in the household. We adjusted the timing of the assessments depending on whether it was a weekday or weekend day, acknowledging that sleep and wake schedules may be somewhat different when the young carers attended school or not on a particular day. On weekdays, push notifications to complete assessments were sent at 7:00 am, 3:00 pm, and 7:00 pm. On weekend days, push notifications were sent at 8:00 am, 3:00 pm, and 8:00 pm. The young carers had a two-hour window to complete each assessment, and a reminder was sent if the survey was not completed within an hour of the initial push notification.

At the end of the week, participants were debriefed and compensated for participation. They were given USD 10 a day for a total of USD 70.00. A total of 341 prompts were sent out, with 260 completed, 8 viewed but not completed, and 73 not viewed or completed. This resulted in a 76.24% compliance rate with 17.33 assessments per young carer.

### 2.2. Participants

Given the exploratory nature of this study and the need to show proof of concept, the age range was kept broad. Young carer inclusion criteria consisted of the following: (1) between the ages of 10 and 19; (2) have a family member living with ALS; (3) participate in some measure of care for a family member; (4) fluent in English; (5) no history of obstructive sleep apnea to avoid confounds related to assessment of sleep quality; and (6) not pregnant. Young carers (*n* = 15, *k* observations = 260) from the southeastern Wisconsin area were recruited and enrolled. Baseline data were available for 11 of the young carers. Of these, most (*n* = 9, 81.82%) were caring for a father with ALS, with the remaining for a mother with ALS (*n* = 2, 18.18%). Participants were aged between 10 and 19 (*M* = 14.55, *SD* = 2.17), 54.55% (*n* = 6) female, 45.45% (*n* = 5) male, 90.91% (*n* = 10) identifying as White/Caucasian, and 9.09% (*n* = 1) Black/African American.

### 2.3. Materials

At the initial session, young carers completed baseline questions about their age, gender, race, and care recipient.

#### 2.3.1. Caregiving and Caregiving Perceptions

At each EMA, young carers first indicated if they had recently engaged in caregiving, answering yes (1) or no (0) to the following question: “Since the last time you filled out a survey, did you do a caregiving task?” If they indicated yes, they were then asked what activities they did, selecting all that applied from the following options, drawn from the Multidimensional Assessment of Caring Activities Checklist [23]: personal/hygiene (e.g., helping a family member undress or bathe), housekeeping/errands/appointments (e.g., helping pay bills or make calls), technology/therapy/medical equipment, basic care (e.g., cooking meals or helping to walk), emotional (e.g., keeping family member company), taking care of siblings, and other.

If they answered yes to a caregiving task, they also reported their perceptions of the caregiving moment, based on a scale adapted from the Young Caregiver of Parents Inventory [24]. On a scale of 0 (not at all) to 100 (very much), young carers read the question stem “When I am doing these care task(s), I” and then reported on the items shown in Table 1. To test if there were subcategories to these caregiving reaction items, we performed an exploratory factor analysis using a varimax rotation to maximize the likelihood of observing unique factors. As can be seen in Table 1, items fell into two factors representing a fulfillment factor (feel good about myself, do not feel alone) and a factor about lack of resources (wish for more support, feeling overwhelmed). Across all moments, the fulfillment items were strongly correlated (*r* = 0.67, *p* < 0.001), with a moderate correlation for the lacking resources items (*r* = 0.44, *p* < 0.001). As such, the pairs of items for each factor were averaged together at each moment to create the perception of caregiving scores.

If the young carers had not recently had a caregiving episode, they reported a set of items related to worry about caregiving. First, they indicated yes (1) or no (0) if they were with the care recipient with the following item: “Are you currently with the family member you provide care to?” If they reported no, they answered on a 0 (not at all) to 100 (very much) scale their level of worry about not being present to deliver care: “How much are you worried about not being there to provide care if they need it?”

#### 2.3.2. Cognitive and Emotional Outcomes

At each EMA, regardless of caregiving activities, the young carers answered questions about their cognitive and emotional states. Items were answered on a 0 (not at all) to 100 (very much) scale and can be found in Table 2. To test if there were subcategories to these cognitive and emotional items, we again performed an exploratory factor analysis with a varimax rotation. As reported in Table 2, items fell into four factors representing feeling discontent (anger, frustration, worry, sadness, lacking energy, and tense), focused (concentrating, not distracted, and happy), distressed (anxious, stressed, and tired), and relaxed (content and calm). Items for each factor were averaged together at each moment to create the cognitive and emotional state scores.

### 2.4. Analytic Plan

Descriptive statistics were used to test Research Question 1. We calculated a percentage of all prompted moments in which a care task was reported as occurring recently. Then, within the data for the assessments in which a caregiving task was reported as occurring, we calculated the percentage of care moments in which each type of care task was reported. Finally, we summed the number of types of tasks during each caregiving moment and reported the frequencies for the number of tasks at each caregiving moment.

Research Question 2 tested the young carers' cognitive and emotional states during a caregiving moment compared to a non-caregiving moment. Multilevel models were employed using the proc mixed command in SAS v. 9.4, as this is a recommended approach to account for the nested nature of the data with observations nested within individuals [25]. Models predicted discontent, focus, distress, and relaxation, with each outcome tested in a separate model. As predictors, caregiving was entered in two ways: (1) A between-person average of all the yes/no responses, thus representing the proportion of moments one engaged in caregiving. It is interpreted as comparing whether those who did more (versus less) caregiving on average over the assessment week had different average levels of cognitive and emotional states. (2) A within-person score that was calculated as the momentary response of whether they were caregiving at that moment with their average levels of caregiving subtracted (i.e., person-mean centered). It is interpreted as whether there is a difference for that young carer in cognitive and emotional states during moments when they are caregiving compared to moments they are not, controlling for their average level of caregiving. Models controlled for the time of day and whether it was a weekend day. We did not control for demographics as we had a number of participants with these data missing. Although we address this as a limitation below, we were not as concerned by not including these variables since the focus of this paper was on the within-person scores, which systematically remove the potential influence of demographic factors when centering around each participant’s average. A random intercept was also modeled to account for the possibility that participants have differing initial levels of cognitive and emotional values. Given the nested nature of the data, the models employed maximum likelihood estimations to handle missing data by calculating likelihood estimates rather than imputing missing data. Finally, as a measure of effect size, we calculated a pseudo *R*^2^ by producing a model-estimated value at each moment and correlating that with the observed value [26].

Research Question 3 tested if perceptions of the caregiving moments during the point of care related to cognitive and emotional states for young carers. The same models used to test Research Question 2 were employed, except now perceptions of fulfillment and lacking resources were used as the predictors. Also, these models only analyzed data during a caregiving moment. These models entered both the between-person (person mean) and within-person (person-mean centered) versions of these variables. Fulfillment and resources were tested concurrently.

Finally, for the exploratory question, we used the same models as Research Question 2, but now tested levels of worry about not being around for the care recipient as a predictor of cognitive and emotional well-being.

## 3. Results

### 3.1. Descriptive Statistics

Across all moments, the young carers reported low to moderate levels of discontent (M = 32.90, SD = 19.80) and distress (M = 40.56, SD = 22.59), and moderate to high levels of relaxation (M = 62.73, SD = 18.62) and focus (M = 67.96, SD = 16.38). In terms of caregiving perceptions, the young carers reported somewhat low levels of lacking resources (M = 23.33, SD = 20.29) and high levels of fulfillment (M = 77.89, SD = 19.66). The intraclass correlations (ICC) for fulfillment suggested that most of the variance was at the between-person level (ICC = 0.84), but that the variance was more evenly distributed across the between-person and within-person levels for lacking resources (ICC = 0.56).

### 3.2. Research Question 1: How Often Do Youth Report Engaging in Care Tasks?

Participants indicated frequent engagement in caregiving (~39% of all assessments), which comes to slightly more than one care episode a day. As reported in Table 3, they most often reported engaging in basic care tasks (occurring in about half of all caregiving episodes), with consistent engagement in emotional, housekeeping, and hygiene tasks as well. Finally, about a third of all caregiving episodes involved doing multiple types of activities.

### 3.3. Research Question 2: Comparing Caregiving and Non-Caregiving Moments

We next tested cognitive and emotional states when caregiving versus not caregiving. As reported in Table 4, at the between-person level, doing more caregiving on average did not significantly predict any of the cognitive and emotional states (ps > 0.07). Similarly, at the within-person level, moments in which a young carer engaged in caregiving did not differ from moments without caregiving on any of the cognitive and emotional states (*p* > 0.07).

### 3.4. Research Question 3: Perceptions of Caregiving Moments

Next, we tested whether perceptions of caregiving moments predicted cognitive and emotional states. As reported in Table 5, at the between-person level, those feeling more fulfilled by their caregiving also reported feeling more discontent on average (*p* = 0.029) but no differences in the other outcomes (ps > 0.15). Those feeling they were lacking resources on average reported more discontent (*p* < 0.001) but no differences in other outcomes (ps > 0.35).

At the within-person level, in a moment when a young carer felt more fulfilled with the caregiving task, that young carer reported less discontent (*p* = 0.032) and more focus (*p* = 0.004) but no differences in distress or relaxation (ps > 0.15) at that moment. In a moment when a young carer felt they were lacking resources during a caregiving task, that young carer reported more discontent (*p* < 0.001) and more distress (*p* < 0.001) but no differences in focus or relaxation (ps > 0.58) at that moment.

### 3.5. Exploratory Question: Non-Caregiving Moments and Well-Being

Finally, we explored cognitive and emotional well-being during non-caregiving moments. This analysis focused on the 84 moments when the young carers were neither delivering care nor with the care recipient. Overall, the young carers reported moderate levels of worrying about not being available to do a care task if needed (M = 54.82, SD = 27.21). As reported in Table 6, at the between-person level, those who worried more on average reported more average distress (*p* = 0.022) but no differences in discontent, focus, or relaxation (ps > 0.05). At the within-person level, the more a young carer worried about not being available to do a care task, the higher their discontent (*p* = 0.016) and distress (*p* = 0.001) were in these moments. There was no statistically significant relationship between momentary worry and focus or relaxation (ps > 0.61).

## 4. Discussion

This study provided an initial exploration into the momentary perceptions of care and the cognitive and emotional states of young carers with a family member living with ALS. Study participants engaged in frequent care tasks, with more than one care episode a day, across care needs. Additionally, we found that simply engaging in a care task was not related to cognitive and emotional states. Rather, we observed how the young carers *perceived* care in terms of fulfillment and lacking resources that mattered. Not only did perceptions of fulfillment and resources vary from one caregiving episode to the next, particularly for lacking resources, these perceptions predicted outcomes. Specifically, caregiving episodes perceived by young carers as more fulfilling predicted less discontent and more focus in those moments of care, while caregiving episodes perceived as lacking in resources predicted more discontent and distress. Prior studies have also identified higher levels of stress in young carers [27], underscoring the need for continued focus on potential stress and distress in care, across moments of care, and across care recipient needs. This mixed picture underscores that the experience of caregiving is not monolithic and includes both positive and negative aspects, as reflected in previous caregiving studies on other disorders [28]. Thus, as much as care needs and tasks vary by illness or injury, it stands that care varies by person and moment of care, as evidenced by these data. Moreover, engaging in care can be very personal and intimate in illnesses that require intense care such as ALS, providing moments in which caregiving may create bonds and a sense of purpose, and other moments in which caregiving is stressful as it competes with other demands or competes with the emotional development and progression of youth.

Indeed, the current study found consistent, daily engagement in care by young carers that is often complex and consuming: one-third of caregiving episodes involved care covering more than one care domain. These data are clear evidence of the role of young carers as more than watching over or peripherally involved in care, but rather deeply engaged in care, as found across care populations and care needs. These data support the need to understand more fully how the type of task and its potential complexity and difficulty illuminate the impact of caregiving for these young carers. Beyond what task was performed, there was clear evidence that the within-person perceptions of care tasks are important. The effect sizes for these models indicate anywhere from ~14 to 50% of the variance in these outcomes. Given that these perceptions varied from one caregiving episode to another, shaping perceptions and providing resources to young carers in caregiving moments represent a clear potential time to intervene. This finding is particularly critical in the US, where young carers have few resources and are not federally recognized compared to the UK, which has clear recognition and additional support for young carers [6]. These data can be used to not only provide targeted support and interventions but also to advocate for the inclusion of youth as caregivers in care policy, highlighting the intensity of care and the moments throughout the day needed for support.

In moments when young carers found the caregiving task fulfilling, they reported less discontent and more focus. This may explain a sense of achievement in care, which is critical not solely for the care experience but reflects similar needs for youth achievement in other aspects of life [29], providing an avenue for developmental and personal growth. Yet at the between-person level, we do see a potentially perplexing finding that those who reported more fulfillment on average generally reported higher discontent. While this may be a suppression effect, it may also highlight the effect that more care, while fulfilling, can also be more difficult. This demonstrates another way in which caregiving may be perceived as complex and mixed, even within-person and care tasks, and points to the need for more support targeting the actual care and the moment of care, as opposed to post-care. Moreover, these data highlight the need for a greater understanding of how care experiences differ across care recipient needs, care tasks, intensity, and experience.

Addressing the lack of resource perceptions, the results were clear in addressing the negative impact of care in moments when young carers feel overwhelmed. These findings highlight the need for both more support in the moment (based on the within-person effects) and also the accumulation of lacking sources (based on the between-person effects). At the between-person level, those who reported more generally that they were lacking resources also reported generally more discontent, perhaps indicating potential unhappiness with life. Prior research has shown not only the need for more support for young carers [30,31] but also the positive effect of resources and support. The current data support the consistent need for resources in care and overall support for the young carer throughout the care experience.

Finally, the data, while exploratory, provided strong evidence of worry across time points including when no care is being provided, adding to existing data identifying worry and young carers [32]. Young carers worry in school and struggle with completing assignments, which is clearly a time when no care is taking place. The current study provides further evidence that the responsibility of care permeates well beyond active caregiving and underscores the need for mental health support at all times, including school-based support and support for the family as a whole. Further, the existence of worry outside care tasks might imply the need for more broad family support, given that worry may stem from the youth feeling unsure whether others are providing needed care in their absence. Thus, education and support for the entire family or household unit may serve to alleviate the worry of young carers. Finally, it may be that the youth assume their care is the best or the most effective, thus worry when not being the one providing the care. This points to a further need to provide mental health support for the youth and wraparound care and support for the family to alleviate worry across family members.

### Limitations

While mental health and school performance outcomes are critical, little attention has been paid to the physical impacts of caregiving, despite clear evidence of caregiving impacting adult caregivers' overall health and well-being [33,34] and some initial data demonstrating the negative impact of caregiving on the sleep patterns for youth carers [35]. Future programming and research must attend to the physical experience of caregiving.

Although we were able to observe a large number of moments and caregiving episodes, the exploratory sample size we drew from was small. This created a few limitations. First, we had some missing data on demographics for the initial group of participants. We opted not to control for these factors as we did not want to lose data and because the focus of this paper was on the importance of momentary perceptions that, through the person-mean centering we performed, systematically separate the moment-to-moment variability (the within-person variable) from the more stable aspects of perception that could be influenced by demographics and personality (the between-person variable). Yet future research may wish to control for demographics and potentially test them as a moderator, testing, for example, whether perceiving a caregiving moment as having fewer resources is more strongly connected to distress for younger carers than older ones or based on gender and race/ethnicity. Unlike adult caregivers who are primarily women, data have long supported that all genders engage in caregiving as youth [36]. Additionally, while young carers are most often identified in programs and research over the age of 15 [37], youth as young as 5 engage in care tasks and take on significant care roles [38]. Yet how perceptions of caregiving vary by gender, age, and/or race/ethnicity for young carers is unknown, as well as whether these perceptions differentially predict outcomes like distress and discontent depending on demographics.

Finally, in our assessment of caregiving tasks, we were able to determine the types of activities the caregiving youth were performing. Yet we do not know about the overall load at any one moment. For example, a young carer may have been reporting a series of caregiving tasks within the same category. Not only might this indicate more load, but some tasks within a category may also be more intense than others. With more data and more fine-grained assessments of what occurred during each caregiving episode, we will be further primed to understand the resources necessary to support young carers.

## 5. Conclusions

As the US moves towards more inclusion and recognition of caregivers across populations, these data can be used to support the critical need to not only acknowledge young carers and the often-intense role they play as caregivers but also that the perception of care varies, potentially affecting well-being, school performance and attendance, and relationships. At the global level, these initial data can be used to inform larger studies of targeted intervention in the moment of care, with the potential to lessen the negative impacts of care and heighten the positive ways in which children and youth engage in care.

## Figures and Tables

**Table 1:** Exploratory factor analysis of reports of how participants felt when caregiving.

Item	Fulfillment Factor	Lack of Resources Factor
Feel good about myself	**0.83**	−0.11
Feel alone	**−0.76**	0.37
Wish I had more help/support	−0.15	**0.90**
Am overwhelmed	−0.22	**0.61**

Note: Items in bold within each column were averaged together to create scores for analyses.

**Table 2:** Exploratory factor analysis of cognitive and emotional feelings at each moment.

Item	Discontent Factor	Focus Factor	Distress Factor	Relaxed Factor
I feel angry.	**0.85**	−0.10	0.15	−0.03
I feel frustrated.	**0.83**	−0.16	0.21	−0.06
I worry about the future.	**0.74**	0.12	0.003	−0.04
I feel sad.	**0.74**	−0.32	0.16	−0.01
I lack the energy needed to complete tasks I want to complete.	**0.69**	−0.09	0.09	−0.12
I feel tense.	**0.63**	−0.27	0.24	0.01
I feel I can concentrate.	−0.07	**0.78**	−0.14	0.15
I feel distracted.	0.35	**−0.63**	0.15	0.02
I feel happy.	−0.01	**0.51**	−0.06	0.28
I feel anxious.	0.10	−0.13	**0.82**	0.02
I feel stressed.	0.55	0.04	**0.66**	−0.19
I feel tired.	0.16	−0.27	**0.49**	0.02
I feel content.	−0.03	0.24	0.25	**0.80**
I feel calm.	−0.08	0.06	−0.15	**0.49**

Note: Items in bold within each column were averaged together to create scores for analyses.

**Table 3:** Types and frequencies of caregiving tasks.

	How Often Completed
Overall Occurrence of Caregiving	
Any Task	39.23% (102/260)
Frequency of Types of Care	
Basic care	50.98% (52)
Emotional	24.51% (25)
Housekeeping	24.51% (25)
Hygiene	22.55% (23)
Siblings	19.61% (20)
Technology	12.75% (13)
Others	4.90% (5)
Frequency of Total Number of Tasks	
1 task	66.67% (68)
2 tasks	14.71% (15)
3 tasks	8.82% (9)
4 tasks	4.90% (5)
5 tasks	1.96% (2)
6 tasks	0.98% (1)

**Table 4:** Unstandardized beta coefficients (standard errors) of caregiving moments and cognitive and emotional outcomes.

	Discontent	Focus	Distress	Relaxed
Random Effects				
Intercept	**391.31** **(161.95)**	**129.19** **(57.67)**	**396.15** **(170.16)**	**154.80** **(79.38)**
Residual	**129.19** **(11.76)**	**167.86** **(15.28)**	**218.70** **(19.93)**	**193.20** **(17.75)**
Fixed Effects				
Intercept	**60.69** **(16.23)**	**38.31** **(10.91)**	**63.00** **(17.11)**	**43.80** **(11.87)**
Weekend	**−3.48** **(1.60)**	2.49 (1.83)	−4.05 (2.09)	**3.94** **(1.96)**
Time of day	**−0.01** **(0.003)**	**0.01** **(0.003)**	**−0.01** **(0.003)**	0.003 (0.003)
Care moment (between-person)	−50.06 (41.28)	48.33 (26.88)	−20.80 (43.10)	38.76 (29.28)
Care moment (within-person)	−1.42 (1.53)	−1.70 (1.75)	−0.68 (2.00)	−3.32 (1.88)
Model Effect				
Pseudo *R*^2^	<0.001	0.061	0.014	0.185

Note: Coefficients in bold are significant at least at *p* < 0.05.

**Table 5:** Unstandardized beta coefficients (standard errors) of perceptions of caregiving and cognitive and emotional outcomes.

	Discontent	Focus	Distress	Relaxed
Random Effects				
Intercept	**84.01** **(42.13)**	**67.49** **(38.48)**	**279.88** **(142.46)**	**292.41** **(158.67)**
Residual	**68.41** **(10.62)**	**122.32** **(18.99)**	**191.92** **(29.90)**	**175.82** **(27.67)**
Fixed Effects				
Intercept	−15.85 (18.67)	37.54 (18.15)	38.21 (33.67)	85.47 (34.14)
Weekend	−3.21 (1.94)	4.55 (2.57)	−3.82 (3.25)	4.71 (3.11)
Time of day	−0.003 (0.004)	**0.01** **(0.01)**	−0.01 (0.01)	−0.005 (0.01)
Fulfillment (between-person)	**0.43** **(0.19)**	0.27 (0.18)	0.11 (0.35)	−0.18 (0.36)
Fulfillment (within-person)	**−0.23** **(0.11)**	**0.43** **(0.14)**	−0.24 (0.18)	0.24 (0.17)
Lack of resources (between-person)	**0.70** **(0.20)**	−0.09 (0.19)	0.16 (0.35)	−0.33 (0.36)
Lack of resources (within-person)	**0.54** **(0.07)**	−0.05 (0.09)	**0.51** **(0.11)**	−0.001 (0.11)
Model Effect				
Pseudo *R*^2^	0.497	0.255	0.139	0.2037

Note: Coefficients in bold are significant at least at *p* < 0.05.

**Table 6:** Unstandardized beta coefficients (standard errors) of worry when not caregiving and cognitive and emotional outcomes.

	Discontent	Focus	Distress	Relaxed
Random Effects				
Intercept	**187.33** **(92.90)**	84.12 (52.89)	**161.64** **(86.41)**	**82.30** **(52.36)**
Residual	**151.38** **(25.70)**	**194.85** **(33.26)**	**171.74** **(29.31)**	**219.84** **(37.38)**
Fixed Effects				
Intercept	**27.88** **(10.77)**	**64.85** **(9.05)**	**37.60** **(10.49)**	**67.42** **(9.32)**
Weekend	−1.77 (3.63)	−0.64 (4.02)	−4.16 (3.85)	1.59 (4.25)
Time of day	**−0.01** **(0.01)**	0.01 (0.01)	**−0.02** **(0.01)**	0.01 (0.01)
Worry (between-person)	0.33 (0.17)	−0.02 (0.12)	**0.37** **(0.16)**	−0.15 (0.13)
Worry (within-person)	**0.36** **(0.15)**	−0.03 (0.17)	**0.53** **(0.16)**	0.09 (0.18)
Model Effect				
Pseudo *R*^2^	0.293	0.014	0.319	0.079

Note: Coefficients in bold are significant at least at *p* < 0.05.

## Data Availability

The datasets presented in this article are not readily available due to technical/time limitations. Requests to access the datasets should be directed to Kavanaugh or Zawadzki.
